# Congenital Hypothyroidism in Neonates of a Tertiary Care Hospital

**DOI:** 10.12669/pjms.335.12986

**Published:** 2017

**Authors:** Adeel Ahmad, Anam Wasim, Shahida Hussain, Muhammad Saeed, Bilal Munir Ahmad, Khalil ur Rehman

**Affiliations:** 1 Adeel Ahmad, M.Phil. Department of Microbiology, University of Health Sciences, Lahore, Pakistan; 2 Anam Wasim, BS (MLT). Department of Microbiology & Molecular Genetics Punjab University, Lahore, Pakistan; 3 Shahida Hussain, M.Phil. Department of Microbiology & Molecular Genetics Punjab University, Lahore, Pakistan; 4 Muhammad Saeed, M.Phil. Department of Microbiology & Molecular Genetics Punjab University Lahore Pakistan; 5 Bilal Munir Ahmad, M.Phil. Pathology Department, Mayo Hospital, Lahore, Pakistan; 6 Khalil ur Rehman, FCPS. Professor of Pathology, Sahiwal Medical College, Sahiwal, Pakistan

**Keywords:** Congenital Hypothyroidism, Incidence, Neonatal Screening

## Abstract

**Objective:**

To determine neonatal congenital hypothyroidism among neonates born in a tertiary care hospital of Lahore Pakistan.

**Methods:**

This cross-sectional study was carried out at Pathology Department of Allama Iqbal Medical College, Lahore in collaboration with Pediatrics and Gynecology & Obstetrics Department, Jinnah Hospital, Lahore Pakistan. A total of 770 babies were included in this study, both male and female. About 2 ml venous blood samples were collected aseptically from the neonates in sterile clotted tube. Serum was separated and serum TSH was determined by ELISA method.

**Results:**

Out of total 770 neonates, 48.9% were female and 51.0% were males with the ratio of 1:1.04. Neonatal congenital hypothyroidisim (TSH, >30 mIU/L), was observed in 0.4% (Frequency, 1:257) nenates, with the incidence rate of 1:257. Female to male ratio of hypothyroid neonates was 2:1. The mode of delivery vise distribution showed, among n=251 neonates born by normal delivery, only a single case of hypothyroidism was detected, and among n=519 neonates delivered by cesarean section, only two neonates were belong to hypothyroidism.

**Conclusion:**

The frequency of Congenital Hypothyroidism is notably higher in pediatric community than reported in most other countries. This result emphasizes the necessity of a nationwide screening program.

## INTRODUCTION

Congenital hypothyroidism (CH) is the most common preventable cause of mental retardation.^1^ CH is defined as thyroid hormone deficiency or defective thyroid function at birth.^2^ CH is classified as permanent CH, a condition of persistent thyroid hormone deficiency which requires prolong treatment, and a transient CH, in which deficiency of thyroid function appears temporarily and then there is recovery. The recovery from transient CH most probably occurs in first few months or years after birth.^3^

In infants it is difficult to diagnose as there are few symptoms. The diagnostic features may include prolonged jaundice, umbilical hernia, macroglossia, feeding difficulty, mottled skin, lethargy, hypothermia, edema, hypotonia, abnormal cry and hypothyroid appearance.^4^

Previously the incidence of CH was in the ratio of 1:2000 to 1:4000 before screening was introduced.^5^ Now this range stands at 1:4000 after congenital screening programs were instituted.^6^ In developing countries, newborn screening (NS) is mandatory. However in countries like Pakistan no Newborn Screening program has been started yet. Therefore, a study was planned to evaluate the incidence of congenital hypothyroidism by measuring the TSH levels in the blood of infants of Pakistani pediatric population so as to recommend guidelines for establishing Newborn Screening.

## METHODS

A total of 770 infants were selected from Pediatrics and Obstetrics& Gynecology Department of Jinnah Hospital, Lahore, Pakistan. A sample of 770 was estimated using winpepi version 11.15 with 95% confidence interval and acceptable level of 3% and assuming proportion of 0.24 of congenital hypothyroidism. The study was carried out in Dept. of Pathology from Jun 2014 to December 2015 after approval of Ethical Review Board of Allama Iqbal Medical College, Lahore. The study population included male and female babies. Neonates less the 48 hours of age, history of taking radioactive iodine by mother, babies with multiple congenital anomalies and premature babies less than 37 weeks of gestation were excluded from the study.

About 2 ml blood was collected from each neonate 2-10 days age. Serum was separated and TSH level was determined by immunoassay technique. TSH level <30mIU/L was considered as reference value. Every test showed hypothyroid state was repeated on same sample, and similar results were obtained.

### Statistical analysis

Data were analyzed statically with the help of SPSS 21.0 software. The demographical data was presented in the form of percentages and frequency. P value was calculated from winpepi ver: 11.15 and.24 is fraction

Sample size calculation for single proportion n = (Z/E)^2^ p (1-p)

Where n = required sample size Z is critical value for two tailed test and E is margin of error P = is proportion from pervious study.

## RESULTS

Out of total 770 neonates screened for hypothyroidism by measuring serum TSH level, n= 377 (48.9%) were female and n=393 (51.0%) were males with a female to male ratio of 1:1.04. Most of pediatric population enrolled in this study belonged to lower socioeconomic class. Five hundred and seventy-one n= 571 (74.2 %) neonates had normal status and n=199 (25.8%) were sick and admitted in pediatrics department.

Three neonates n=3 (0.4%) (Frequency, 1:257) were proved to be hypothyroid. The mode of delivery was also noted and results revealed that n=251(32.5%) babies were born by normal delivery and n=519 (67.4%) cases were delivered by cesarean section. One neonate was found to be hypothyroid in normal delivery group and two were from C-section group. Although statistically there was no significant association between mode of delivery and CH, however higher frequency among C-section cases was seen, which may be associated with some hidden pathologies. There was non-significant association between male and female for CH, its same as 2:1 are male and female and series of 770 with 3 CH chi-square is significant at.000.

## DISCUSSION

Congenital Hypothyroidism is known as one of the most common reason of mental retardation, more specifically related to mental impairment and growth retardation in infants. Therefore, it is essential to screen new born for CH during early days of life so that proper treatment may be started for hypothyroid babies.

We found a frequency of 1:257 of hypothyroidism in new born babies. The overall CH incidence reported worldwide is 1 in 4000. Our study has found a much higher frequency of CH. This finding is in agreement with other studies from our region.^7^ Limited studies were conducted on screening of CH in Pakistan. Ghaffor et al., from Pakistan screened 1357 newborns for the blood TSH level and reported the CH incidence 2 out of 1357 cases.^8^ Afroze et al., investigated the screening of CH in Pakistani pediatric population in 2008. In this hospital based study, only 10 babies were diagnosed with congenital hypothyroidism. They reviewed the data of 2008 over 10 months, in order to estimate the actual incidence rate. The final incidence rate they reported were 1 in 1600 live births.^9^

**Fig.1:**
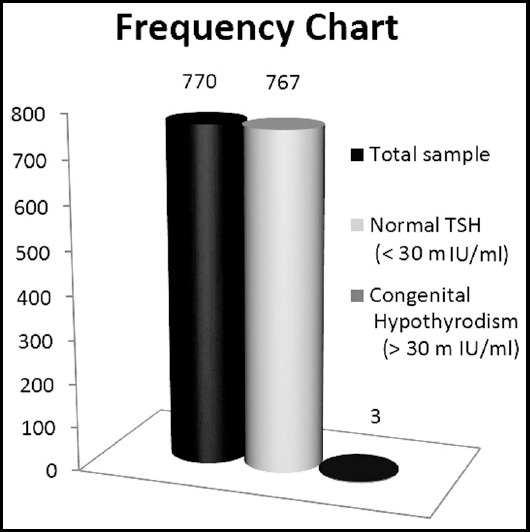
Frequency graph of Total, Normal and Congenital Hypothyroidism of population after measuring TSH Level.

In a study from India, Devi et al reported a frequency of 1 in 2481 births.^10^ The higher incidence may be due to racial or geographic factors and warrants for making the availability of cost effective therapy and nationwide implementation of active screening programs based on TSH assay. An Iranian study has reported an incidence of 1 in 914.^11^

Gender seems to be an important risk factor for development of CH. The female to male ratio of hypothyroid babies was found to be 2:1. Previous studies were in agreement with our findings.^12^ Therefore, there is possibility that females have increased risk.^13^ Several Canadian, European and Australian studies have reported a female to male ratio for true CH cases as 2:1.^14^-^16^

We also investigated the role of type of delivery and TSH level among infants. In our study association was found between mode of delivery and congenital hypothyroidism. Out of 251 normal deliveries only one case and 519 cesarean section two cases were found to be hypothyroid Table-I. Similar findings were reported by another author, who was concluded that babies delivered by cesarean section are significantly more likely to have elevated TSH levels> 5 mIU/L as compared to those normal delivery cases. However, another study showed no association between the mode of delivery and abnormal TSH level. In another study observed elevated TSH level more in forceps extraction as compared to C-section, which contradicts findings of current study.^17^

**Table-I:** Type of delivery and TSH level Cross tabulation.

*Type of Delivery*	*TSH*		*Total*

	*Normal (< 30 mIU/L)*	*Congenital Hypothyroidism (> 30mIU/L)*	
Normal delivery	250	01	251
C-section	517	02	519

Total	767	03	770

Pearson’s chi-square = 0.001, P = 0.978.

Congenital hypothyroidism infants are normal at birth showing few signs and symptoms. This factor prevents early diagnosis. In developing countries, much less data is available about incidence of CH due to lack of screening programs. Presently, neonatal screening of CH is considered an essential parameter in the perspective of primary child health care such as immunization, oral dehydration and breast feeding. The findings of our study suggest the need to establish neonatal screening programs in Pakistan. However, a larger population based study may determine the incidence of CH as well correlation of normal delivered baby and delivered by cesarean section more accurately.

Primary CH is the most common cause of this condition. However, transient cases, which may be caused by maternal antithyroid medication, exposure to topical iodine, maternal iodine deficiency or excess, maternal TSH receptor blocking antibodies, medications (dopamines, steroids), or prematurity (<30 weeks), may also occur and are not rare. All these cases should be treated as CH for the first three years of life by taking into account the risks of mental retardation. A re-evaluation after the age of three years is needed in such patients.

### Authors’ Contribution

***AA:*** Sample analysis and manuscript write up

***AW:*** Designed the study, Sample collection and sample performing

***SH:*** Conceived and designed the experiment, statistical analysis.

***MS:*** Sample collection and sample performing, manuscript write up.

***BMA:*** Statistical analysis of data, interpretation of data and *Reviewer of manuscript*.

***KR:*** Over all supervision, interpretation of results and manuscript writing.

## References

[1] Büyükgebiz A (2013). Newborn screening for congenital hypothyroidism. J Clin Res Pediatr Endocrinol.

[2] Klett M (1997). Epidemiology of congenital hypothyroidism. Expert Clin Endocrinol Diab.

[3] Bona G, Bellone S, Prodam F, Monzani A Etiology of Congenital Hypothyroidism. Thyroid Diseases in Childhood. Springer.

[4] Karamizadeh Z, Saneifard H, Amirhakimi G, Karamifar H, Alavi M (2012). Evaluation of congenital hypothyroidism in Fars province, Iran. Iran J Pediatr.

[5] Ford G, LaFranchi SH (2014). Screening for congenital hypothyroidism:a worldwide view of strategies. Best Pract Res Clin Endocrinol Metabol.

[6] Deladoëy J, Van Vliet G (2014). The changing epidemiology of congenital hypothyroidism:fact or artifact?. Expert Rev Endocrinol Metabol.

[7] Manglik AK, Chatterjee N, Ghosh G (2005). Umbilical cord blood TSH levels in term neonates:a screening tool for congenital hypothyroidism. Indian Pediatr.

[8] Ghafoor F, Mohsin SN, Mukhtar S, Younas S, Hussain W (2013). Newborn screening for congenital hypothyroidism in a public sector hospital. Pak J Med Res.

[9] Afroze B, Humayun KN, Qadir M (2008). Newborn screening in Pakistan—lessons from a hospital-based congenital hypothyroidism screening programme. Ann Acad Med Singapore.

[10] Devi ARR, Naushad S (2004). Newborn screening in India. Indian J Pediatr.

[11] Ordookhani A, Minniran P, Najafi R, Hedayati M, Azizi F (2003). Congenital hypothyroidism in Iran. Indian J Pediatr.

[12] Ali S, Memon SH, Baluch GH (2017). A study of nonspecific symptoms in hypothyroidism. Medical Channel.

[13] Lorey FW, Cunningham GC (1992). Birth prevalence of primary congenital hypothyroidism by sex and ethnicity. Hum Biol.

[14] Medda E, Olivieri A, Stazi MA, Grandolfo ME, Fazzini C, Baserga M (2005). Risk factors for congenital hypothyroidism:results of a population case-control study (1997–2003). Eur J Endocrinol.

[15] Deladoey J, Be-langer N, Van Vliet G (2007). Random variability in congenital hypothyroidism from thyroid dysgenesis over 16 years in Quebec. J Clin Endocrinol Metab.

[16] Jones JH, Mackenzie J, Croft G, Beaton S, Young D, Donaldson M (2006). Improvement in screening performance and diagnosis of congenital hypothyroidism in Scotland 1979-2003. Arch Dis Child.

[17] Seth A, Sekhri Τ, Agarwal A (2007). Effect of perinatal factors on cord blood thyroid stimulating hormone levels. J Pediatr Endocrinol Metab.

